# Comprehensive Review on Thermoplastic Polyurethane: Applications in Wound Healing and Smart Healthcare

**DOI:** 10.3390/biomimetics11070491

**Published:** 2026-07-13

**Authors:** Karuppasamy Nandhini, Nae Yoon Lee

**Affiliations:** Department of BioNano Technology, Gachon University, 1342 Seongnam-daero, Sujeong-gu, Seongnam-si 13120, Gyeonggi-do, Republic of Korea

**Keywords:** TPU, antibacterial, wound healing, skin regeneration, tissue engineering, smart healthcare systems

## Abstract

Thermoplastic polyurethane (TPU) is an important polymer widely used in biomedical applications owing to its flexibility, strength, low toxicity, and biocompatibility. The TPU structure is classified into two types—soft and hard segments—which can be easily modified to achieve the desired mechanical and biological properties, making TPU an ideal material for wound healing and smart healthcare systems. Unlike conventional thermoset polyurethanes, TPU has a segmented architecture with soft and hard domains that undergo microphase separation. This unique structure provides an excellent balance of elasticity, toughness, flexibility, and processability, allowing TPU to better mimic the mechanical behavior of soft biological tissues, making it suitable for wound healing and smart healthcare applications. TPU-based dressings provide a moist environment, allow oxygen to pass through, and protect wounds from bacterial infection. Compared with traditional materials, TPU offers greater elasticity, durability, and patient comfort. In addition, TPU plays a key role in wearable sensors, electronics, and real-time monitoring devices. TPU-based smart materials can detect pH, temperature, and moisture levels to monitor wound conditions and overall patient health. This review highlights the potential of TPU as a promising material for wound healing and numerous smart healthcare applications.

## 1. Introduction

Chronic wounds are a significant global health problem. They affect millions of people worldwide and place a considerable clinical and economic burden on healthcare systems. The aging population and the increasing prevalence of diabetes, obesity, and peripheral vascular disease have resulted in a steady rise in chronic wounds, including diabetic foot ulcers, venous leg ulcers, arterial ulcers, and pressure ulcers [1,2]. Among these, diabetic foot ulcers are one of the most serious complications of diabetes and are often associated with persistent infection, delayed healing, lower-limb amputation, prolonged hospitalization, and increased mortality. Wound infections further impair tissue regeneration, complicate treatment, and increase the need for antimicrobial therapy [2]. Chronic wounds are a major clinical and economic burden because they require long-term treatment, frequent outpatient visits, prolonged hospitalization, and extensive use of healthcare resources [3]. Recent estimates suggest that the management of chronic wounds accounts for approximately 2–5% of total healthcare expenditure in several European and Australian healthcare systems [1,4]. These challenges highlight an urgent need for advanced wound dressing materials that can accelerate tissue repair, prevent infection, and enable real-time monitoring of the wound microenvironment for personalized wound management.

Thermoplastic polyurethane (TPU) is a versatile class of elastomeric polymers that has gained significant attention in biomedical and healthcare applications owing to its excellent mechanical flexibility, biocompatibility, durability, and physicochemical properties [5,6]. The molecular structure of TPU consists of both soft and hard segments, which provide structural flexibility, elasticity, toughness, and mechanical strength. These properties enable TPU to mimic the mechanical behavior of soft biological tissues, making it suitable for advanced biomedical applications, including wound dressings, wearable sensors, and smart healthcare systems [7,8]. In tissue engineering applications, porosity is a key design parameter for TPU-based scaffolds. Interconnected porous structures support cell infiltration, nutrient and oxygen (O_2_) diffusion, waste removal, and extracellular matrix (ECM) deposition. However, pore size and porosity must be carefully optimized because highly porous scaffolds exhibit reduced tensile strength and dimensional stability, whereas insufficient porosity limits cell penetration and tissue integration [8,9]. This review provides a comprehensive overview of the structure, physicochemical properties, fabrication methods, and recent applications of TPU in wound healing and smart healthcare.

**Figure 1:**
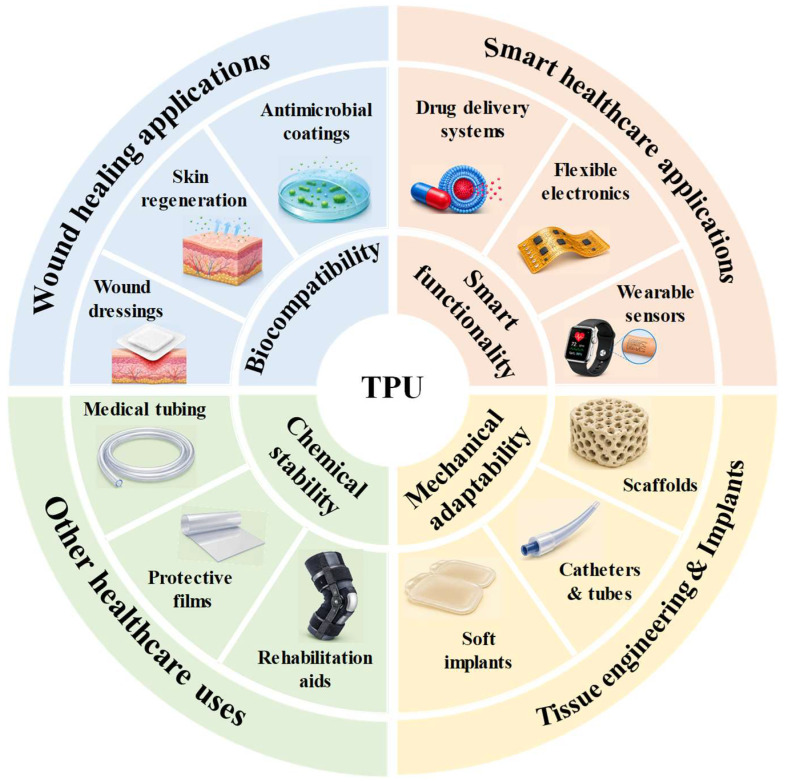
Schematic overview of TPU functional properties and biomedical applications.

In recent years, TPU-based materials have encouraged researchers to investigate their biomedical applications, including advanced wound healing, antibacterial coatings, skin regeneration, catheters, biosensors, tissue engineering, artificial organs, drug delivery systems, wearable sensor devices, and other healthcare applications, as shown in Figure 1 [10]. Among these applications, TPU has emerged as a promising material for advanced wound healing owing to its ability to provide a favorable environment for tissue repair and regeneration [11,12]. An effective wound dressing should maintain a moist environment, allow O_2_ permeation, prevent microbial infection, and support cell adhesion and proliferation. TPU-based wound dressings exhibit excellent flexibility, breathability, transparency, moisture retention, mechanical stability, and biocompatibility, which are essential characteristics for effective wound management [13]. Moreover, TPU can be readily functionalized with antibacterial agents, bioactive molecules, nanoparticles, and natural polymers to inhibit microbial growth and enhance its therapeutic performance [14]. Furthermore, the development of multifunctional TPU systems has expanded their applications in smart healthcare technologies. TPU-based smart materials can exhibit self-healing, shape-memory, antibacterial, and biodegradable properties, making them suitable for wearable sensors, electronic skin, medical devices, and real-time health monitoring systems [14,15]. Therefore, TPU-based materials represent a promising platform for the development of next-generation biomedical and healthcare technologies. The increasing demand for advanced wound care materials has driven the development of polymeric biomaterials that can accelerate healing, prevent infection, and enhance patient comfort [16].

Traditional wound dressings, such as cotton gauze, are often limited in their ability to maintain a moist environment, provide adequate antimicrobial protection, and conform to irregular wound surfaces. In contrast, TPU-based materials offer improved flexibility, breathability, transparency, and moisture management, which are key characteristics for maintaining a conducive wound healing environment. Moreover, TPU can be readily fabricated into nanofibers, films, hydrogels, and porous scaffolds using electrospinning, solvent casting, and three-dimensional (3D) printing processes, further extending its applications in tissue engineering and regenerative medicine [17]. Another significant advantage of TPU is its ability to incorporate functional nanomaterials, antimicrobial agents, and therapeutic compounds. The incorporation of nanoparticles, carbon-based nanomaterials, metallic nanostructures, and bioactive molecules into TPU matrices improves antimicrobial activity, electrical conductivity, mechanical stability, and drug release performance [18]. Therefore, multifunctional TPU composites are increasingly being investigated for smart wound dressings that can monitor wound conditions, detect infections, and deliver therapeutics in a controlled manner.

The rapid development of wearable electronics and flexible biosensors has increased the importance of TPU in smart healthcare technologies. TPU acts as an ideal substrate for flexible electronic devices, such as strain sensors, temperature sensors, and physiological monitoring systems, owing to its excellent stretchability and skin compatibility [10,13]. TPU-based smart materials can continuously monitor vital signs, human motion, sweat biomarkers, and wound healing progression, enabling personalized healthcare and real-time medical diagnostics. The integration of TPU with emerging technologies, such as the Internet of Things (IoT), artificial intelligence (AI)-assisted diagnostics, and wireless healthcare platforms, has further expanded its potential in advanced smart healthcare applications [19].

However, long-term biostability, controlled biodegradability, large-scale manufacturing, and regulatory approval remain important considerations for the clinical translation of TPU-based healthcare products. Accordingly, current research is focused on developing multifunctional TPU composites with improved biological performance, sustainability, and smart responsiveness. This review provides a comprehensive overview of TPU, including its structure, physicochemical properties, fabrication methods, and recent applications in wound healing and smart healthcare. Particular focus is placed on recent advances in TPU-based nanocomposites, wearable sensors, antimicrobial dressings, and smart healthcare systems, along with the current challenges and future outlook for this rapidly growing field.

### 1.1. Importance of Wound Healing and Smart Healthcare

TPU has attracted considerable attention for wound healing and smart healthcare owing to its excellent flexibility, biocompatibility, mechanical strength, breathability, and facile processability [1,6]. Smart wound management and intelligent healthcare systems have gained increasing importance because of the growing demand for high-end healthcare materials [4]. Wound healing is a fundamental biological process that is essential for restoring the integrity and function of tissues damaged by injuries, burns, surgery, or chronic diseases such as diabetes. Effective wound care is important for preventing infection, reducing inflammation and scarring, and promoting faster tissue regeneration [14,18]. Traditional wound healing materials primarily provide physical protection. In contrast, modern wound healing materials are designed to actively facilitate the healing process by providing antimicrobial activity, moisture retention, O_2_ permeability, and real-time monitoring capabilities. Meanwhile, smart healthcare has emerged as an advanced approach that integrates wearable devices, biosensors, nanotechnology, AI, and flexible biomaterials to enable continuous health monitoring and personalized treatment [19]. Smart healthcare technologies facilitate early disease diagnosis, remote patient monitoring, rapid diagnosis, and improved treatment efficiency. In wound management, smart systems can monitor parameters such as pH, temperature, moisture, and bacterial infection [14,15]. Timely medical intervention can greatly improve patient outcomes, and TPU has emerged as a promising material for wound healing and smart healthcare applications because of its flexibility, durability, transparency, and compatibility with functional nanomaterials and electronic components [11]. TPU-based materials are widely investigated for smart wound dressings, wearable sensors, drug delivery systems, tissue engineering scaffolds, and flexible healthcare devices. These multifunctional properties make TPU an important material for the next generation of biomedical and healthcare technologies [15].

### 1.2. Limitations of Traditional Wound Healing Methods and Healthcare Systems

Although significant advances have been made in wound care materials and healthcare technologies, traditional wound healing methods and healthcare systems still have many limitations. Traditional wound healing primarily relies on the use of gauze, cotton, bandages, and basic wound dressings [6,14]. These materials are commonly used to cover wounds, absorb exudate, and protect wounds from external contamination. However, they primarily serve as passive barriers and do not actively promote the different stages of the wound healing process. A major drawback of conventional dressings is their limited ability to maintain an optimal moisture balance. A dry wound environment can delay epithelialization, whereas excessive moisture may lead to maceration of the surrounding skin and delay the healing process. Traditional wound dressings often fail to maintain a moist wound environment, which is essential for efficient wound healing and tissue regeneration. Another important limitation is the pain and secondary tissue injury associated with dressing removal. Gauze and cotton dressings may adhere to newly formed granulation tissue [14,17]. This adhesion can damage the healing tissue, increase inflammation and bleeding, and slow the healing process during dressing changes. Traditional wound dressings are also limited in their ability to prevent and detect infections. These systems cannot monitor wound biomarkers, including pH, temperature, O_2_ levels, glucose, and bacterial load. Consequently, infections and abnormal wound healing may be identified only at a late stage, resulting in delayed intervention and potential complications [10,13]. Additionally, traditional healthcare systems often depend on periodic hospital visits and visual wound assessments by clinicians. This approach is time-consuming, costly, and may fail to detect the early signs of infection or delayed healing because they are not always visibly apparent. Therefore, there is an urgent need for advanced wound dressing materials that can provide moisture balance, antibacterial activity, controlled drug release, real-time wound monitoring, and atraumatic removal [20]. Functional polymer-based dressings, including TPU-based systems and smart dressings, offer a promising approach to overcoming these limitations by responding to wound conditions and improving personalized wound care.

## 2. Overview of TPU

TPU is an important class of polyurethane elastomers that combines the processability of thermoplastics with the flexibility and toughness of rubber-like materials. Its structure can be tailored by modifying the soft segment, hard segment, chain extender, and filler composition. Because of this tunable nature, TPU has become highly attractive for biomedical devices, wound dressings, wearable sensors, and point-of-care testing (POCT) systems. This review discusses TPU in terms of its chemistry, structure–property relationships, physicochemical properties, surface modification strategies, and role in smart healthcare platforms.

### 2.1. Introduction of TPU

TPU is a unique block copolymer containing urethane linkages and has found widespread applications in chemistry and biomedicine. Since it was first synthesized by the German professor Otto Bayer in 1937, TPU has attracted significant attention in various industrial and biomedical applications [21,22]. TPU is a linear, segmented polyurethane that softens and melts upon heating and can be reprocessed into different shapes. This thermoplastic behavior distinguishes TPU from many cross-linked thermoset polyurethanes. It can be processed by solvent casting, melt extrusion, electrospinning, 3D printing, and film formation. These fabrication methods make TPU suitable for preparing membranes, nanofibers, porous scaffolds, flexible sensors, and wound dressing substrates. In biomedical applications, TPU is valued because it provides a balance of elasticity, mechanical stability, chemical resistance, and biocompatibility [23]. TPU materials have been explored for tissue engineering, artificial organs, wound healing, medical catheters, surgical sutures, drug delivery systems, and bioflexible electronics [24]. The strong interest in TPU mainly arises from its adjustable molecular design. Researchers can employ a range of polyols, diisocyanates, chain extenders, and fillers to engineer TPUs with tailored levels of softness, stiffness, degradation behavior, antimicrobial activity, conductivity, and self-healing ability. TPU is particularly useful for wound healing and smart healthcare because it can mimic the mechanical properties of soft skin tissue, withstand body motion, and facilitate the integration of sensing and therapeutic elements [25,26]. Therefore, TPU is not merely a passive polymeric support but also a functional platform for next-generation smart wound dressings and POCT devices.

### 2.2. Structure and Compositions of TPU

**Figure 2:**
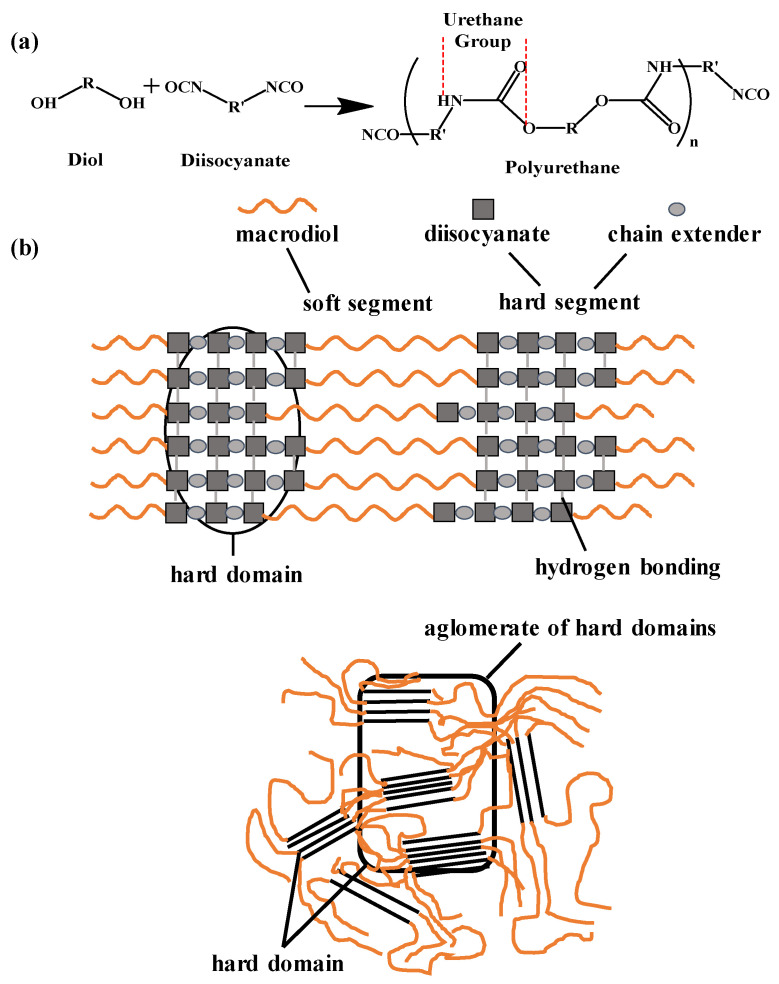
(**a**) Chemical structure of PU. and (**b**) Schematic representation of the domain structure of PUs, [32]; reprinted with permission from [21], copyright 2024 ACS.

TPU is typically composed of three major components: (i) long-chain polymer diols or polyols, (ii) diisocyanates, and (iii) short-chain chain extenders, such as diols or diamines. As shown in Figure 2, the soft segments are composed of long-chain polyols, whereas the hard segments consist of diisocyanates and chain extenders [27,28]. The polymer chain consists of alternating soft and hard segments, resulting in a segmented block copolymer structure. The soft segments are responsible for the elasticity, flexibility, and ability of TPU to maintain its mechanical properties at low temperatures. Common soft segments include polyether polyols, polyester polyols, polycarbonate polyols, polycaprolactone (PCL)-based polyols, and bio-based polyols derived from renewable resources such as castor oil and soybean oil [29]. Polyether-based TPU generally exhibits excellent hydrolytic stability and flexibility, whereas polyester-based TPU provides superior mechanical strength but may be more susceptible to hydrolytic degradation. Polycarbonate-based TPUs are often considered for biomedical applications because they offer improved stability and mechanical performance [30]. Diisocyanates and chain extenders are used to form the hard segments. These components impart strength, stiffness, physical cross-linking, and thermal stability. Microphase separation is induced by the polarity difference between the rigid hard and the flexible soft segments. Within this structure, the hard domains serve as reinforcement, whereas the soft domains provide elasticity. Therefore, the final behavior of TPU depends on the proportion and distribution of the soft and hard segments. In general, a higher soft-segment content confers greater flexibility and elongation, whereas a higher hard-segment content provides greater strength, stiffness, and dimensional stability. This structure–property relationship allows TPU to be tailored for a wide range of biomedical and healthcare applications [31].

### 2.3. Physicochemical Properties of TPU

The physicochemical properties of TPU make it highly suitable for wound healing and smart healthcare applications. Key properties of TPU include elasticity, high tensile strength, tear resistance, abrasion resistance, chemical resistance, transparency, breathability, moisture permeability, and excellent processability [21]. Depending on its composition, TPU typically exhibits a tensile strength of 20–60 MPa, an elongation at break of 300–700%, and a Young’s modulus ranging from 10 to 100 MPa, making it suitable for flexible biomedical and healthcare applications [12]. Table 1 summarizes the mechanical properties of medical-grade TPU, which fall between those of highly compliant soft tissues (e.g., skin and skeletal muscle) and mechanically robust connective tissues (e.g., tendons). Compared with native skin, TPU exhibits substantially greater extensibility and mechanical durability, while its moderate stiffness and tensile strength allow it to withstand repeated deformation without structural failure. This combination of mechanical compliance and robustness makes TPU particularly attractive for dynamic biomedical devices subjected to continuous bending, stretching, and compression [33,34]. These properties can be tailored by varying the chemical composition and the ratio of soft to hard segments. The segmented molecular structure of TPU provides a unique combination of elasticity, tensile strength, and toughness. The soft segments impart flexibility and allow large deformations, whereas the hard segments act as physical cross-links and reinforce the polymer network [35]. This structure–property relationship enables TPU to exhibit mechanical properties comparable to those of soft biological tissues while remaining durable under repetitive deformation. Therefore, TPU is particularly suitable for wound dressings, wearable sensors, and tissue engineering applications.

Flexibility and mechanical stability are critical for wound dressing applications because the dressing must adhere to the skin without restricting movement [36]. TPU has excellent elasticity and toughness, allowing the material to bend, stretch, and recover during daily activities. Moisture and gas permeability are also important because wounds require O_2_ exchange and an optimal moisture balance to promote healing. TPU films and electrospun TPU nanofiber membranes can be designed to serve as protective barriers while allowing controlled moisture vapor transmission. Another important property is biocompatibility. Medical-grade TPU has been used in many healthcare devices because of its relatively low cytotoxicity when in contact with tissues [37]. However, the biological response depends on the TPU composition, additives, degradation products, surface chemistry, and sterilization method. For long-term applications, biostability, oxidative stability, and hydrolytic stability must be carefully considered. TPU can also be modified to impart additional functions, such as antibacterial activity, electrical conductivity, shape-memory behavior, self-healing ability, and controlled drug release. These advanced properties can be achieved by incorporating functional modifiers and additives, such as silver nanoparticles (AgNPs), zinc oxide (ZnO), graphene oxide, titanium dioxide, carbon nanotubes (CNTs), MXene, polydopamine, chitosan, gelatin, conductive polymers, dynamic covalent bonding units, and hydrogen-bonding units, into the TPU matrix [5,38]. Such modifications enhance the performance of TPU in advanced wound healing and smart healthcare applications.

**Table 1:** Mechanical properties of medical-grade TPU.

Authors	Material	Young’s Modulus (MPa)	Tensile Strength (MPa)	Elongation at Break (%)	Ref.
Wang, 2006	Tendon	500–1500	50–150	10–20	[39]
Morrow et al., 2010	Skeletal muscle	0.01–0.10	0.1–0.5	40–60	[40]
Aisling Ní Annaidh et al., 2012	Human skin	1–83	13–30	35–115	[41]
Hao-Yang Mi et al., 2013	Medical-grade TPU	10–100	20–60	300–700	[42]

### 2.4. Surface Modifications of TPU

TPU possesses favorable bulk properties; however, its surface often requires modification to improve biological performance. Surface modification is important because the material surface is the first point of contact between a biomaterial and the body. Cell attachment, protein adsorption, bacterial adhesion, blood compatibility, wettability, and drug release behavior are influenced by the surface properties of wound dressings and biomedical devices [43]. Common surface modification strategies include plasma treatment, chemical grafting, coating, blending, immobilization of bioactive molecules, and incorporation of nanoparticles. Plasma treatment can increase surface energy and introduce functional groups, thereby improving hydrophilicity and cell interactions [44]. Chemical grafting is often accomplished through covalent bonding between the activated TPU surface and bioactive molecules. The TPU surface can be functionalized with reactive groups, such as hydroxyl, carboxyl, and amine groups, through plasma treatment or chemical oxidation. These groups can subsequently be linked to biomolecules using coupling agents, such as carbodiimide (EDC/NHS) chemistry or silane coupling agents [45,46]. These approaches enable the functionalization of TPU surfaces with molecules such as heparin, peptides, chitosan, collagen, hyaluronic acid, and antimicrobial agents. Surface coatings can provide antibacterial, anti-inflammatory, and antifouling properties while also improving adhesion to biological tissues and enhancing wound-site retention. TPU can also be combined with nanomaterials, such as AgNPs, ZnO, graphene oxide, CNTs, MXene, and conductive polymers, to enhance antibacterial activity, mechanical strength, electrical conductivity, and sensor performance [38]. Surface modification of TPU for wound dressing applications can improve moisture management, inhibit bacterial growth, promote fibroblast proliferation, and enable the controlled release of therapeutic agents. However, careful optimization of surface modification strategies is required. Excessive modification can reduce flexibility, cause cytotoxicity, alter degradation behavior, or compromise long-term stability. Therefore, the optimal strategy is to enhance surface biocompatibility without compromising the intrinsic mechanical properties of TPU.

### 2.5. Role of TPU in POCT Systems

Point-of-care testing (POCT) is a diagnostic and monitoring approach that can be performed at or near the patient’s point of care instead of in a traditional centralized laboratory. POCT systems are valuable in wound healing and smart healthcare because they provide rapid insights into physiological and biochemical conditions, enabling timely clinical decision-making [47]. TPU is a promising material for POCT platforms because of its flexibility, stretchability, lightweight nature, skin compatibility, and ease of processing into thin films, fibers, membranes, and wearable substrates. These features make TPU suitable for wearable applications such as flexible biosensors, wearable patches, smart bandages, microfluidic devices, and skin-interfaced diagnostic platforms [48]. TPU enables the integration of electrodes, conductive nanomaterials, colorimetric indicators, enzymes, nanoparticles, and wireless modules. TPU-based POCT systems have been developed to monitor wounds by detecting pH, temperature, moisture levels, pressure, O_2_ levels, glucose, lactate, inflammatory markers, and bacterial infection. These data are particularly valuable because the wound microenvironment in chronic wounds often changes before clinical signs become visible [49]. Real-time monitoring can help clinicians identify infection, delayed healing, and abnormal inflammation at an earlier stage. TPU is also mechanically compatible with the skin. An effective POCT patch should conform to body movements without peeling, cracking, or causing discomfort. TPU can maintain close contact with the skin and wound surface while protecting the integrated electronic components. Therefore, TPU serves as both the structural support and the functional interface of smart healthcare devices [50].

### 2.6. Scope of This Review

This review highlights the role of TPU in wound healing and smart healthcare applications. It first describes the general structure and composition of TPU, the relationships among the soft segments, hard segments, microphase structure, and material properties, and then discusses the key physicochemical properties of TPU relevant to wound dressings, biomedical devices, and wearable healthcare systems. The review also highlights surface modification strategies for enhancing the biological and functional performance of TPU. Special attention is given to TPU-based wound dressings, antibacterial composites, electrospun membranes, drug delivery systems, wearable sensors, and POCT platforms. The discussion covers emerging functions such as self-healing, electrical conductivity, shape-memory behavior, and real-time wound monitoring. Finally, the review addresses current challenges and future directions, including long-term biostability, controlled biodegradability, antimicrobial durability, scalable fabrication, sensor reliability, patient comfort, and clinical translation. This review aims to provide a clear understanding of the role of TPU in supporting the development of next-generation wound healing and smart healthcare technologies by bridging TPU chemistry and biomedical function.

## 3. Applications of TPU-POCT

TPU-based materials have gained significant attention in POCT and smart healthcare systems owing to their flexibility, stretchability, biocompatibility, and ease of modification. TPU can be readily processed into nanofibers, films, hydrogels, sponges, patches, coatings, and wearable sensors. Furthermore, TPU can be combined with bioactive molecules, conductive fillers, and antimicrobial agents for applications in wound protection, advanced wound healing, tissue regeneration, and real-time health monitoring.

### 3.1. Antibacterial and Wound Healing Systems

TPU-based antibacterial and wound healing systems are primarily designed to protect wounds, prevent bacterial infection, and promote faster tissue repair (Table 2). In most studies, TPU is used as a flexible supporting matrix, whereas functional components such as porphyrin, MXene, gelatin, citrate, gallic acid, silver nanowires (AgNWs), and antibacterial polymers provide additional therapeutic functions [5]. For instance, TPU nanofiber dressings can mimic the ECM and provide a large surface area for cell attachment. These dressings can be functionalized with antibacterial agents to eliminate bacteria and reduce the risk of infection. TPU can also provide intelligent functions, such as light-triggered antibacterial activity, Joule heating-assisted drug release, wet-tissue adhesion, self-healing behavior, and exudate management [19]. These properties make TPU suitable for applications in infected wound management, soft tissue repair, surgical sutures, and smart wound dressings. Overall, the role of wound dressings has shifted from passive wound coverage to active wound treatment in TPU-based wound healing systems.

**Figure 3:**
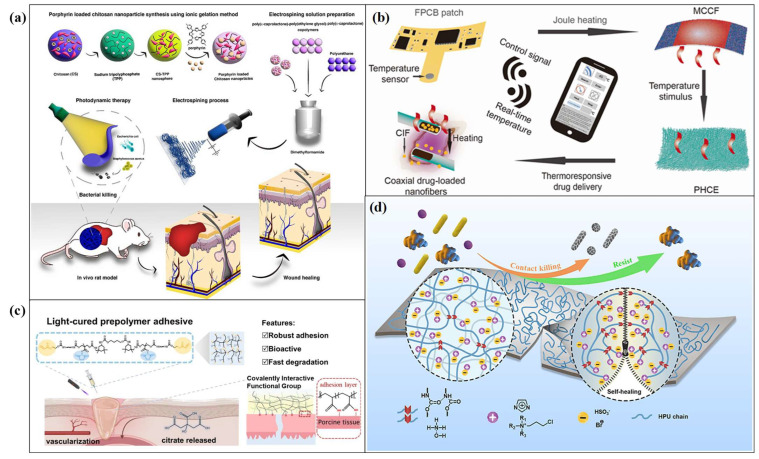
(**a**) TPU-based porphyrin-containing nanofibrous mats exhibiting photosensitive antibacterial activity and enhanced cutaneous wound healing performance; reprinted with permission from [51], copyright CC BY 4.0. (**b**) MXene/TPU hybrid fabrics for smart wound management, real-time monitoring, and thermoresponsive drug delivery; reprinted with permission from [52], copyright 2024 ACS. (**c**) Bioactive citrate-based PU tissue adhesive for rapid wound healing and accelerated tissue regeneration; reprinted with permission from [54], copyright CC BY 4.0. (**d**) Antibacterial, antifouling, and self-healing nanocellulose/PU composites for biomedical catheter applications; reprinted with permission from [56], copyright 2024 Elsevier.

**Figure 4:**
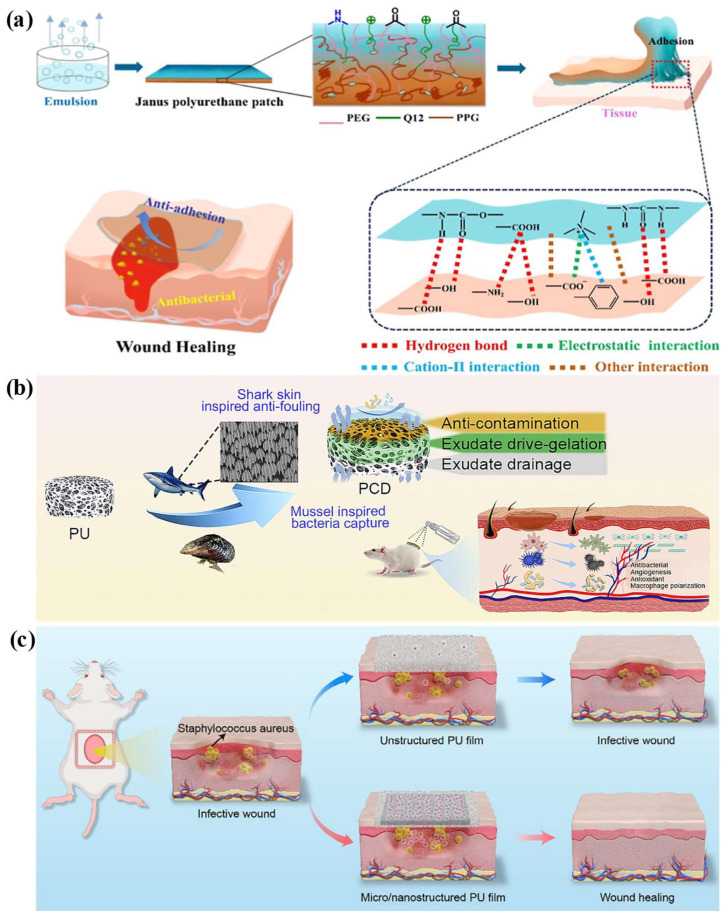
Schematic illustrations showing the wound healing outcomes of advanced TPU-based wound dressings. (**a**) Janus PU adhesive patches; reprinted with permission from [57], copyright 2024 ACS. (**b**) Biomimetic shark skin- and mussel-inspired PU hydrogel sponges; reprinted with permission from [61], copyright 2025 Elsevier. (**c**) Ultrafast laser micro/nanostructured PU dressings; reprinted with permission from [62], copyright 2025.

Saghebal et al. [51] developed a polyurethane-based nanofibrous mat loaded with porphyrin photosensitizers for antibacterial wound healing. The nanofibrous structure is important because it mimics the ECM and provides a large surface area for cell attachment and drug release (Figure 3a). In this system, porphyrin serves as the active antibacterial component. Upon irradiation with red light, porphyrin generates reactive oxygen species (ROS) that damage bacterial membranes and reduce the survival of *Staphylococcus aureus* (*S. aureus*) and *Escherichia coli* (*E. coli*). The scaffolds containing chitosan- and porphyrin-loaded nanoparticles also facilitate more effective release of the active agent. In a rat wound model, the dressing promoted faster wound closure and enhanced tissue regeneration. This work demonstrates that TPU-based nanofibers can be converted into active antibacterial dressings by incorporating light-responsive agents. The major limitations are the requirement for an external light source to achieve the antibacterial effect and the limited light penetration into deep and irregular wounds [51]. Cheng et al. described the use of an MXene/TPU hybrid fabric for thermoresponsive drug delivery and smart wound care [52]. Unlike conventional dressings, this system combines therapy and monitoring on a single platform. The MXene layer acts as a Joule-heating unit, whereas the medication is carried by a TPU-based nanofiber layer. Heating of the MXene layer is induced by applying a low voltage, which triggers the thermoresponsive layer to release the medication (Figure 3b). In addition, the integrated flexible electronic circuit can monitor wound temperature. This capability is valuable because an increase in wound temperature may indicate inflammation or infection. In vitro and in vivo studies demonstrated greater antibacterial activity and faster wound healing than conventional gauze. This work represents a significant advancement in the development of next-generation smart wound treatment systems. Yildirim et al. fabricated a double-layer electrospun dressing from TPU and gelatin. The design concept was to mimic the two main layers of the skin [53]. TPU provides flexibility and mechanical stability, whereas gelatin provides a more cell-friendly surface. The natural bioactive agent Hypericum perforatum oil was incorporated to improve the antibacterial and wound-healing properties of the dressing. O_2_ plasma treatment was performed to improve the adhesion between the TPU and gelatin layers. The dressing exhibited appropriate water vapor transmission and fluid absorption properties, which are necessary to maintain a moist wound environment without excessive exudate accumulation. Antibacterial activity was observed against both Gram-positive and Gram-negative bacteria. In vitro wound healing assays showed that the plasma-treated dressing was biocompatible and stimulated cell migration. This study highlights the potential of bilayer TPU dressings for wound healing. However, the fabrication process is relatively complex because it involves electrospinning, natural oil incorporation, and plasma treatment. Li et al. demonstrated a citrate-based polyurethane adhesive for rapid tissue sealing and improved wound healing [54]. Tissue adhesives offer the advantage of closing wounds without causing additional tissue damage and have emerged as a useful alternative to sutures. The hydrophobic PCL segments in this material facilitate the removal of water from the wet tissue surface and improve adhesion under physiological conditions. The adhesive forms strong interfacial interactions and mechanical interlocking. A significant advantage of citrate is its bioactive role. During degradation, citrate can promote angiogenesis, which is essential for supplying O_2_ and nutrients to the healing tissue (Figure 3c). The adhesive also demonstrated hemostatic ability, biodegradability, and good biocompatibility. This work demonstrates the potential of TPU-based adhesives to simultaneously provide mechanical wound closure and biological support for wound healing. However, the synthesis and optimization of wet-adhesion properties may be more challenging than the fabrication of simple films and fiber dressings. Wang et al. synthesized a gallic acid-based waterborne polyurethane with self-healing, antimicrobial, and shape-memory properties. Gallic acid is a plant-based polyphenol with antibacterial and antioxidant properties. In this polyurethane system, gallic acid participates in a dynamic phenol–carbamate network [55]. These dynamic bonds can dissociate and reform upon heating, allowing the material to repair small cracks and damage. The addition of Cu-MOF enhances the antimicrobial activity against *Escherichia coli* (*E. coli*) and *Staphylococcus aureus* (*S. aureus*). This type of material is particularly valuable for wound-related healthcare applications because damaged coatings and films can lose their protective function during body movement. A self-healing polyurethane coating can maintain its structural integrity for a longer period. It may be useful in wearable healthcare devices, antibacterial coatings, and flexible medical materials. The main limitation is that self-healing typically requires thermal activation, which must be carefully controlled in biomedical applications. Zhao et al. developed amphiphilic nanofibrillated cellulose/PU composites for catheter-related applications [56]. Catheters are in direct contact with body fluids and can readily develop bacterial colonization and biofilm formation, resulting in chronic infection and device failure. In this study, amphiphilic nanofibrillated cellulose and PU were combined through dynamic interactions, such as hydrogen bonding and electrostatic interactions. The composite exhibited antibacterial, antifouling, and self-healing properties. It also maintained good mechanical recovery after repeated stretching and demonstrated high cell viability. The self-healing ability in artificial urine is particularly important for urinary catheter applications because the material must function in a wet and chemically complex environment (Figure 3d). This study demonstrates the potential of TPU-based composites for applications beyond wound dressings, particularly in infection-resistant medical devices. However, additional long-term in vivo studies are required before clinical translation. Guo et al. introduced a Janus polyurethane adhesive patch with two distinct surfaces [57]. One side exhibits strong wet adhesion and can attach to tissue, whereas the other exhibits anti-adhesive behavior and prevents unwanted adhesion to gloves, clothing, and surrounding tissue. This asymmetric design is advantageous because conventional double-sided adhesives may adhere to healthy tissue and cause secondary damage during removal. The PU patch also contains quaternary ammonium salt groups that impart antibacterial activity. The hydrophilic components help absorb interfacial water and improve wet-tissue adhesion. In animal studies, the Janus patch promoted wound closure and improved healing in infected wounds (Figure 4a). This study is important because it simultaneously addresses two practical challenges: infection control and selective adhesion. The main challenge is maintaining stable asymmetric surface properties during storage, handling, and clinical use. Xu et al. developed a phase-separated porous PU nanocomposite containing AgNW networks for wireless bioelectronics [58]. Although this work does not describe a direct antibacterial dressing, it is highly relevant to smart wound monitoring. The porous PU structure allows conductive networks to form with a very low filler content, thereby enhancing stretchability while reducing stiffness. The material maintained its electrical performance under large strains, which is important for skin-mounted and implantable devices because the skin continuously moves and stretches. When integrated with near-field communication technology, the system enabled wireless power and data transmission. In wound care, such conductive TPU platforms can be used for continuous monitoring of temperature, pH, humidity, and biomarkers. This capability helps clinicians assess wound status without the need for frequent dressing removal. The main limitation is that bioelectronic systems require device integration, power management, and long-term biosafety evaluation. Wang et al. developed a high-strength bio-based PU elastomer for surgical sutures, which require high tensile strength, flexibility, and good biocompatibility to maintain wound closure during healing [59]. In this study, bio-based polycarbonate soft segments and asymmetric hard segments were used to improve the mechanical performance of the material. The resulting elastomer exhibited high tensile strength, toughness, and fracture energy. It also demonstrated self-healing and recyclability under heat treatment. Biological studies confirmed good biosafety and biodegradability. When used as a surgical suture in mice, the material promoted wound healing. This study is significant because it demonstrates that TPU-based materials can be designed not only as wound dressings but also as wound closure devices. The bio-based design also supports the development of sustainable biomedical materials. However, additional studies are needed to compare its long-term performance with that of commercial sutures under clinically relevant conditions.

Ding et al. reported a TPU-based medical adhesive for soft tissue wound repair with good biocompatibility, flexibility, and adhesive strength [60]. Soft tissue adhesives should bond strongly to wet tissue, accommodate body movement, and avoid causing irritation. TPU-based adhesives are promising because the tunability of the soft and hard segments enables an optimal balance between elasticity and strength. Compared with rigid adhesives, flexible PU adhesives can reduce stress concentration at the wound site and improve patient comfort. For wound healing, these adhesives could help seal small cuts, protect delicate tissue, and reduce the need for sutures. The key requirements include strong adhesion in wet environments, nontoxic degradation, and stable performance during body movement. This application area is important because effective wound closure depends not only on mechanical sealing but also on adhesive properties that promote a favorable healing microenvironment. Zhang et al. developed a biomimetic polyurethane hydrogel sponge for infected wounds with high levels of exudate. The design was inspired by shark skin and mussel adhesion (Figure 4b). The hydrophobically modified PU sponge acts as a bacterial barrier and helps prevent surface contamination [61]. The dopamine-augmented chitosan hydrogel layer has been shown to effectively absorb wound exudate and trap microorganisms. Furthermore, the porous structure of the sponge facilitates efficient drainage of excess exudate, minimizing tissue maceration and reducing the potential for bacterial growth. In infected wound models, the dressing reduced inflammation, promoted angiogenesis, increased collagen deposition, and improved wound healing compared with conventional dressings. This work demonstrates that TPU-based dressings can simultaneously manage infection and fluid balance. The main limitation is that multilayer biomimetic structures may require careful manufacturing control to maintain consistent wettability and drainage behavior. Jing et al. used ultrafast laser micro/nanostructuring to improve the anti-infective performance of polyurethane wound dressings [62]. Commercial PU films are flexible, transparent, and breathable but generally exhibit limited intrinsic antibacterial activity. In this study, laser direct writing created micro/nanostructures on the PU surface. These structures substantially increased the drug-loading capacity without altering the bulk properties of the PU film. Clindamycin-loaded microstructured PU exhibited clear antibacterial activity against *Staphylococcus aureus* (*S. aureus*). In a rat model of infected wounds, the dressing inhibited infection and promoted wound healing (Figure 4c). This is an attractive approach because it relies on physical surface modification and does not require complex chemical synthesis. It may also be scalable for the fabrication of advanced medical dressings. However, the long-term drug release profile and the potential effects of laser processing on mechanical durability must be carefully evaluated. Wang et al. [63] synthesized a Janus nanofiber dressing for the sequential treatment of acute wounds. The design consists of a hydrophilic inner layer loaded with *Atractylodes macrocephala* extract and a hydrophobic outer layer of PAN/TPU nanofibers loaded with fusidic acid. Upon contact with wound exudate, the inner layer dissolves rapidly and releases anti-inflammatory components during the early inflammatory phase. The outer layer acts as a physical barrier and provides sustained antibacterial protection to prevent reinfection. The rationale behind this spatiotemporal design is that wound healing requires different therapeutic interventions at different stages. The dressing demonstrated in vitro antibacterial activity and reduced M1 macrophage polarization. In vivo studies demonstrated an increased wound healing rate, enhanced re-epithelialization, improved collagen maturation, and increased angiogenesis. This work represents an advanced TPU-based dressing that combines external protection with internal biological regulation [63].

### 3.2. Skin Regeneration and Tissue Engineering

TPU has been extensively investigated for skin regeneration and tissue engineering applications owing to its elasticity, mechanical stability, and inherent cell compatibility (Table 3). Tissue engineering scaffolds should support cell adhesion, cell growth, nutrient transport, and tissue formation. TPU can be combined with nanofillers, hydrogels, ECM components, and bioactive molecules to enhance its biological and mechanical properties. TPU-based nanocomposites can also be used for skin regeneration because they support fibroblast and keratinocyte growth while providing a flexible surface for wound repair. TPU provides mechanical support and withstands repeated body movement, making it suitable for muscle and tendon tissue engineering applications. TPU-based hydrogels and scaffolds can also be functionalized with peptides and ECM components to enhance cell-material interactions and guide tissue regeneration. Thus, TPU is not only a useful wound dressing material but also a scaffold platform for the reconstruction of damaged skin, muscle, tendon, and bone tissues.

**Figure 5:**
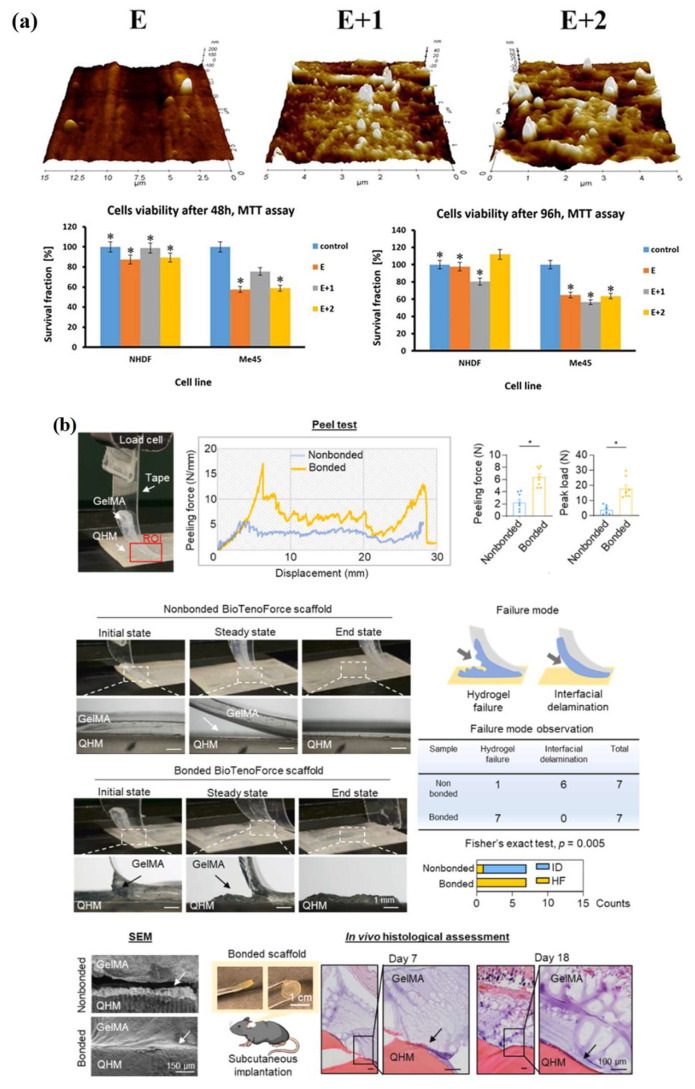
TPU-based biomaterials for skin regeneration and tissue engineering. (**a**) Cytocompatible TPU-based nanocomposites assesses by MTT assay after 48 h and 96 h of incubation in NHDF and Me45 cells; reprinted with permission from [64], copyright 2023 ACS Omega, CC BY 4.0. (**b**) TPU-based BioTenoForce scaffolds for tendon repair and load-bearing tissue regeneration. White and black arrows indicate the interface between the GelMA hydrogel and QHM elastomer. * *p* <0.05; reprinted with permission from [68], copyright 2024 Elsevier, CC BY-NC-ND 4.0.

Mrowka et al. [64] synthesized TPU-based nanocomposites by incorporating halloysite nanotubes (HNTs) into the polymer matrix. This study is important for skin regeneration because the material was designed for application after skin cancer surgery, where the scaffold should promote the growth of normal skin cells without stimulating residual tumor cells. The incorporation of HNTs improved the mechanical properties of TPU, including flexibility and elongation at break, which are essential for skin-contact materials because the skin is soft and continuously moves during healing. The biological results showed lower toxicity toward healthy keratinocytes and fibroblasts than toward cancer cells. This selective cellular response suggests that TPU–HNT nanocomposites may serve as a supportive regenerative platform for postsurgical skin repair (Figure 5a). The underlying mechanism mainly depends on nanofiller reinforcement, improved surface morphology, and enhanced cell-material interactions. However, inflammatory cytokine expression was observed; therefore, the HNT content and surface properties should be optimized before clinical application. Akkurt Yildirim et al. [65] developed electrospun TPU nanofibrous scaffolds doped with clinoptilolite (CLN) for skeletal muscle tissue engineering. Skeletal muscle requires a scaffold that is soft, flexible, and able to support repeated mechanical deformation. TPU provides elasticity, whereas CLN enhances the biological performance of the scaffold. The electrospun nanofibrous structure resembles the fibrous ECM and provides a large surface area for cell attachment. The study showed that cell adhesion and proliferation increased with the addition of CLN, suggesting that the mineral filler enhanced cell attachment. The findings also indicated that the mineral filler improved the cell-friendly nature of TPU. The mechanical results demonstrated tunable Young’s modulus values, which are beneficial because muscle tissue requires scaffolds with controlled stiffness. The underlying mechanism is mainly related to improved surface chemistry, possible hydrogen-bonding interactions, and enhanced cell-material contact. However, higher CLN loading may cause particle agglomeration and reduce structural uniformity; therefore, the filler content must be carefully controlled. Giubertoni et al. [66] investigated changes in hydrogen bonding within TPU during mechanical deformation using rheological two-dimensional infrared spectroscopy (Rheo-2D IR). Although this study is not directly related to tissue engineering scaffolds, it provides valuable insights into the mechanical behavior that makes TPU a promising material for regenerative applications. TPU contains soft segments that provide elasticity and hard urethane segments that form hydrogen bonds. During stretching, these hydrogen bonds rearrange, and weaker bonds may break. This molecular reorganization is responsible for the Mullins effect and explains the stress-softening phenomenon during cyclic loading. For tissue engineering, this mechanism is important because scaffolds used for skin, muscle, tendon, and bone tissues must tolerate repeated body movements without sudden failure. This study provides molecular-level evidence that the mechanical performance of TPU is strongly governed by hydrogen-bond dynamics. These findings can guide the design of future TPU scaffolds with improved fatigue resistance, elasticity, and long-term mechanical stability.

Yuvan et al. [67] reported bioactive PU–poly(ethylene glycol) diacrylate (PEGDA) hydrogels for tissue engineering applications. This work addresses an important limitation of many synthetic scaffolds, namely that they provide good mechanical control but limited biological signaling. In this study, hydrogels with tunable mechanical properties were developed using photocurable PU and PEGDA. The hydrogel network can bind water and create a soft environment similar to the natural ECM. More importantly, the system can be functionalized with peptides and proteins to promote cell adhesion. These biological molecules provide binding sites that enable human cells to adhere to and survive within the hydrogel. The mechanism is based on UV photocrosslinking, which generates a hydrogel network capable of supporting cell-material interactions. This approach is valuable for tissue engineering because it combines the strength and tunability of PU with the hydration and biocompatibility of PEG-based hydrogels. Although the platform is highly adaptable, further optimization is required before clinical translation [67]. Huang et al. [68] engineered an ECM-functionalized load-bearing tendon substitute, termed BioTenoForce, for the treatment of large-to-massive tendon defects. Tendon repair is challenging because the regenerated tissue must withstand high tensile forces while forming an organized collagen-rich matrix. In this design, the PU elastomer provides long-term mechanical support and slow degradation, whereas the tendon ECM supplies tendon-specific biological cues. This dual strategy is important because mechanical strength alone cannot drive regeneration, whereas biological cues alone cannot support large tendon loads. The scaffold exhibited strong interfacial bonding, good suture retention, biocompatibility, and support for the tendon differentiation of human adipose-derived stem cells (Figure 5b). In animal models, the tendon substitute integrated with the host tissue and facilitated the formation of aligned tendon-like tissue while minimizing disorganized scar tissue formation. The mechanism is based on the combination of biomechanical support and ECM-guided tissue regeneration. The main limitation is the complexity of fabrication and the potential variability of ECM-derived materials. Hu et al. developed a polysiloxane-based PU composite double-layer dressing with antimicrobial and antifouling properties [69]. Although this study is discussed under wound dressings, it is also relevant to skin regeneration because an ideal skin repair material must protect the wound while maintaining a favorable healing microenvironment [72]. The dressing contains two functional layers. The outer polysiloxane–PU layer is hydrophobic, transparent, flexible, and mechanically robust, providing protection against liquid penetration and external contaminants. The inner PU pressure-sensitive adhesive layer contains cationic and zwitterionic components. The antimicrobial activity is attributed to the cationic groups, which inhibit protein adsorption and biofouling. PEG segments enhance hydrophilicity and water retention. This combination supports adhesion, exudate management, bacterial inhibition, and patient comfort. Therefore, the underlying mechanism is based on bilayer protection, controlled moisture management, antimicrobial activity, and antifouling behavior. However, additional in vivo and clinical studies are required to confirm its long-term regenerative performance. Karahaliloglu et al. [70] fabricated TPU–oleic acid membranes for guided bone regeneration. Guided bone regeneration requires a barrier membrane that prevents soft tissue invasion while allowing bone-forming cells to attach and regenerate the defect site. Pure TPU provides good flexibility and mechanical strength; however, its hydrophobic surface can limit cell adhesion. In this work, oleic acid was incorporated into TPU, and the surface was further modified by alkali treatment. The treatment reduced the water contact angle and produced a more hydrophilic, nanotextured surface [73]. The modified surface promoted early cell adhesion and exhibited antibacterial activity against *Escherichia coli* (*E. coli*) and *Staphylococcus aureus* (*S. aureus*). The underlying mechanism is mainly based on improved wettability, nanoscale surface roughness, oleic acid-mediated bioactivity, and antibacterial surface properties. However, in vivo guided bone regeneration studies are still needed. Kordbacheh et al. [71] designed a piezoelectric scaffold based on PCL/TPU/barium titanate/cellulose nanocrystal composites for bone tissue engineering. Bone is a naturally piezoelectric material, and its piezoelectric properties regulate bone cell activity and tissue regeneration. To mimic this behavior, barium titanate was incorporated as the piezoelectric component, whereas TPU was incorporated to provide elasticity and flexibility within the PCL-based scaffold. Cellulose nanocrystals enhanced the dispersion of barium titanate, increased hydrophilicity, and reinforced the scaffold. A porous, interconnected structure was fabricated by gas foaming and salt leaching, thereby avoiding the use of toxic solvents and supporting cell infiltration. The scaffold generated measurable electrical output under dynamic culture conditions and enhanced cell adhesion, viability, and proliferation. It integrates structural porosity, mechanical elasticity, and mechanoelectrical stimulation into a single platform. This design is promising for bone regeneration because it provides both physical support and bioelectrical cues. Further studies should focus on the long-term degradation behavior and the optimal filler concentration [71].

### 3.3. Real-Time Physiological Monitoring Systems

TPU-based wearable sensors are important for real-time physiological monitoring because TPU can comfortably adhere to the skin and accommodate body movement without mechanical failure (Table 4). In these systems, TPU is usually combined with conductive and responsive materials such as MXene, graphene, AgNWs, CNTs, conductive polymers, hydrogels, and carbonized fibers. The sensors can convert strain, pressure, humidity, temperature, and biological signals into measurable electrical signals. Consequently, they can be applied to respiration monitoring, sleep monitoring, human motion tracking, ECG recording, posture analysis, facial expression recognition (FER), and human–machine interaction. The major sensing mechanisms include piezoresistive resistance changes, conductive network deformation, humidity-induced proton transport, crack propagation, triboelectric signal generation, and deep-learning-assisted signal classification. TPU-based sensors are soft, flexible, and comfortable for long-term use, making them more suitable for continuous healthcare monitoring than rigid sensors. In summary, TPU-based POCT systems integrate wound care, tissue regeneration, and real-time monitoring into a healthcare platform. This capability makes TPU a promising material for next-generation smart wound healing and personalized healthcare devices.

**Figure 6:**
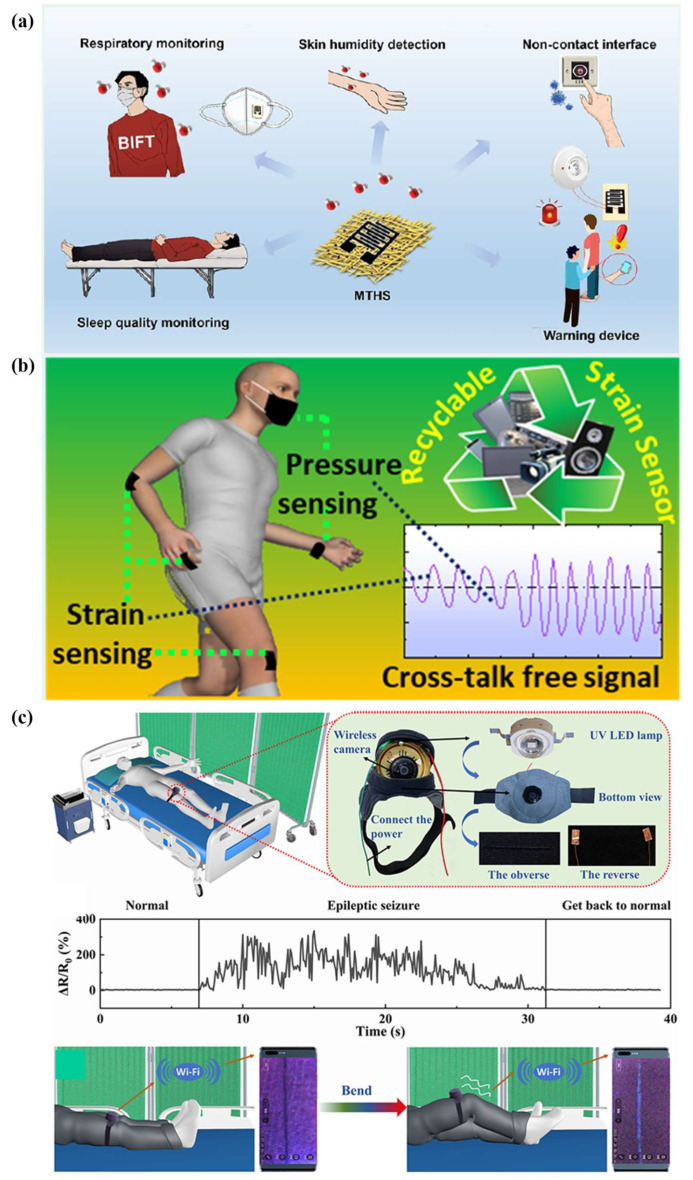
TPU-based wearable sensing platforms for real-time physiological monitoring and smart healthcare applications. (**a**) MXene/TPU nanofiber humidity sensors for respiratory monitoring, skin moisture detection, sleep quality assessment, and contactless sensing; reprinted with permission from [74], copyright 2023 ACS. (**b**) TPU/graphene strain and pressure sensors for motion monitoring with cross-talk-free signal output; reprinted with permission from [75], copyright 2023 ACS. (**c**) Fluorescent TPU-based yarn strain sensors for monitoring body movements and epilepsy-related physiological signals. The red dashed line indicates the enlarged region of the wearable sensing device, and the green dashed line highlights the sensing area; reprinted with permission from [76], copyright 2024 ACS.

**Figure 7:**
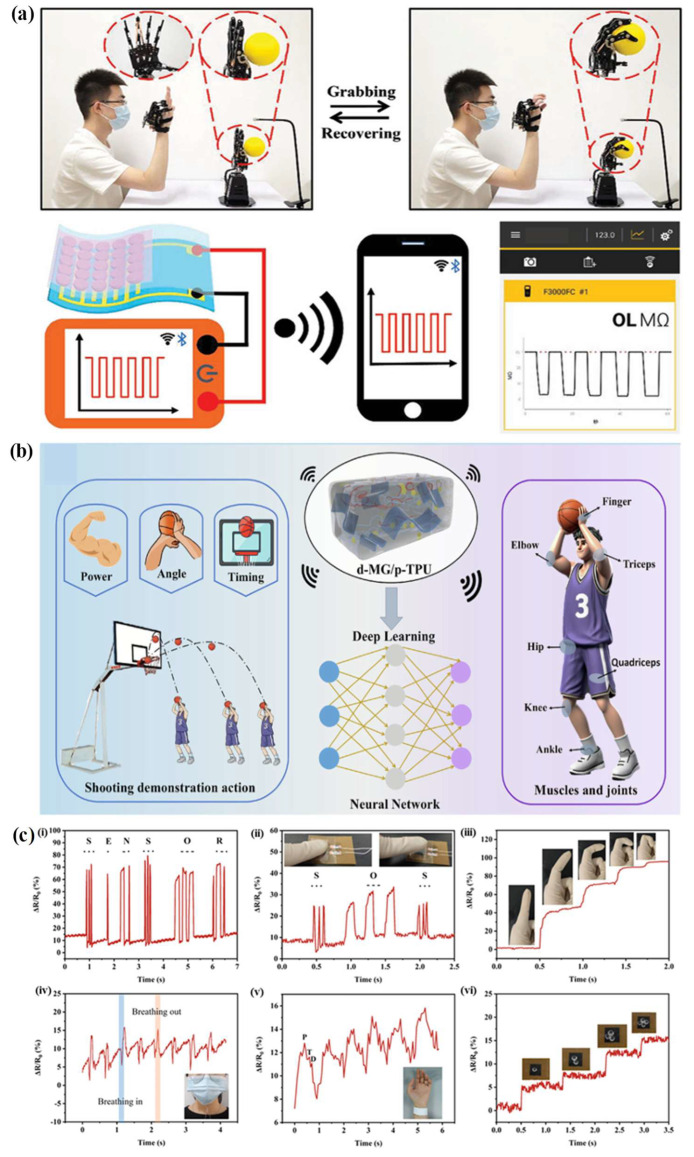
(**a**) TPU-based flexible wearable sensor for human–machine interaction through real-time motion detection and wireless signal transmission; reprinted with permission from [78], copyright CC BY 4.0. (**b**) TPU-based flexible wearable sensor integrated with deep learning for real-time physiological monitoring and intelligent analysis of human motion during basketball shooting; reprinted with permission from [80], copyright 2024 Elsevier. (**c**) Multifunctional TPU pressure sensor for monitoring physiological signals and human activities, including finger movement, respiration, pulse detection, and gesture recognition. The letters S, E, N, S, O, and R represent the sequential pressure pattern used to demonstrate the sensor response. Subsub-figures (**i**–**vi**) show the corresponding sensing responses for different physiological signals and human activities; reprinted with permission from [82], copyright 2024 Elsevier.

Ding et al. [74] fabricated a wearable humidity sensor by integrating Ti_3_C_2_Tx MXene nanosheets with TPU nanofiber membranes. In this design, TPU serves as a flexible and breathable substrate, whereas MXene provides abundant hydrophilic surface functional groups that interact with water molecules. The porous nanofibrous structure can rapidly absorb and desorb moisture from human breath and skin. The sensing mechanism is mainly attributed to the Grotthuss proton transport mechanism, in which adsorbed water molecules form continuous hydrogen-bonded networks that facilitate proton migration (Figure 6a). The electrical response was rapid and could be recorded in real time as the humidity increased during respiration and sweating. The sensor can be used for noninvasive sleep monitoring, breathing analysis, skin moisture detection, and contactless sensing. The main advantages are high sensitivity, a rapid response, and good wearing comfort. However, MXene oxidation may compromise long-term stability; therefore, protective strategies are required for practical healthcare applications. Haridas et al. [75] reported a flexible TPU/graphene sensor for strain and pressure monitoring. TPU provides flexibility and recyclability, whereas graphene forms a conductive network within the polymer matrix. When the material is compressed or stretched, the spacing between graphene particles changes, leading to a change in electrical resistance. This percolation-based conductive network enables the device to detect both strain and pressure signals while minimizing cross-talk. The sensor can monitor both small and large human movements, such as eye blinking, finger bending, and elbow movement (Figure 6b). An important aspect of this work is its sustainability because the sensor can be recycled multiple times with only a small reduction in sensing performance. This feature makes the material attractive for wearable healthcare devices by helping to reduce electronic waste. However, its sensitivity is moderate compared with that of sensors employing advanced microstructured surfaces and crack-based designs. Ai et al. [76] developed a highly stretchable TPU/tetraphenylethylene (TPE)-plied yarn sensor for human health monitoring. TPU and TPE were used to prepare flexible fluorescent fibers, and conductive layers were introduced to provide electrical sensing capability. The sensor operates through crack propagation and conductive network variation during stretching. When the yarn is stretched, cracks open in the conductive layer, causing changes in electrical resistance. At the same time, fluorescence visualization enables direct observation of strain changes. This dual optical-electrical response is useful because it provides both measurable electrical signals and visual feedback. The sensor can monitor pulse, heart rhythm, respiration, lip movements, and joint movements (Figure 6c). It also exhibits a wide strain-sensing range and excellent cycling stability, making it suitable for wearable textiles and personalized health monitoring. The main limitation is the highly complex fabrication process, which includes wet spinning, bead structure formation, and in situ polymerization. Jung et al. [77] developed strain-insensitive outdoor wearable electronics using a thermally robust nanofibrous radiative cooler and a stable conductor. Although this work is not a conventional wound-monitoring platform, it is highly relevant to real-world wearable healthcare because such devices are often used under sunlight and during body movement. The radiative cooler reduces heat accumulation by reflecting sunlight and emitting thermal radiation. At the same time, the strain-insensitive conductor maintains stable electrical performance when the device is stretched. This combination enables the wearable system to collect physiological signals even under harsh outdoor conditions. The material design addresses two common challenges in wearable healthcare: signal distortion caused by movement and thermal discomfort resulting from sunlight exposure. Therefore, this approach can support long-term outdoor health monitoring. The main limitation is the complexity of device fabrication and the potentially higher material cost, particularly when liquid metal conductors are used. Liu et al. demonstrated bioinspired, self-healable antibacterial electronics based on MXene nanosheets [78] and a dynamic polyurethane elastomer. The sensor was inspired by the structure of human skin, in which a protective layer, a microstructured sensing layer, and an electrode layer function together. The MXene-coated microdome structure amplifies pressure signals, and the piezoresistive mechanism converts pressure into changes in electrical resistance. The PU elastomer contains reversible hydrogen bonds, oxime-carbamate bonds, and copper ion coordination bonds, which impart self-healing and recyclability (Figure 7a). These dynamic interactions are important because wearable sensors can be damaged by stretching, scratching, and repeated use. The antibacterial property also improves safety during prolonged skin contact. The sensor exhibited ultra-high sensitivity, a rapid response, and a broad pressure detection range, making it suitable for electronic skin (e-skin), healthcare monitoring, and human–machine interaction. However, the synthesis is relatively complex, and the self-healing process may require thermal activation. Sun et al. [79] fabricated a low-cost flexible pressure sensor by depositing AgNWs and single-layer graphene (SLG) onto a polyurethane sponge. The 3D porous PU sponge provides the composite with compressibility and elastic recovery, whereas AgNWs and graphene form conductive pathways throughout the sponge skeleton. The sponge pores deform under externally applied pressure, thereby altering the contact between conductive pathways and generating a piezoresistive response. This sensor can detect finger movements, human activities, and changes in applied pressure. The simple dip-coating process yields a low-cost system that is amenable to large-scale fabrication. The high-density composite sponge exhibited excellent reproducibility and durability during repeated compression cycles. This work is valuable for wearable pressure sensing, rehabilitation monitoring, and artificial skin applications. However, the sensing performance is highly dependent on the pore size and the uniformity of the conductive coating; therefore, optimization of the pore structure is necessary to achieve higher sensitivity.

Cao et al. developed MXene/GO-based modified TPU flexible sensors integrated with deep learning for basketball shooting posture monitoring [80]. In this system, plasticized TPU provides stretchability, and MXene/GO-DST forms a H-bond dominated conductive network. During body movement, the conductive network changes and produces resistance signals. These signals are collected from different body parts such as fingers, wrists, elbows, knees, and ankles during shooting motions (Figure 7b). A convolutional neutral network is then used to classify whether the shooting posture is standard or incorrect. This work shows the TPU-based sensors can move beyond simple signal detection and become intelligent monitoring systems. The sensor has a wide strain range, fast response, good cyclic stability and high posture recognition accuracy. However, challenges remain including MXene stability issues and the complexity of integrating sensing hardware with deep learning models [80]. Krishna Rajeev et al. reported a flexible conductive polymer reinforced PU foam for ECG monitoring in real-time. The PU foam was reinforced by polyaniline, multiwall carbon nanotubes (MWNTs) and ZnO to create a conductive polymer network [81]. This network makes the material work as a dry electrode patch that collects electrical signals from the heart. The main advantages of this system is that it can reduce the need for conventional Ag/AgCl gel electrodes, which may cause skin irritation, dehydration and signal degradation during long-term use. The fabricated PU composite electrodes were successfully used for R-peak detection. This renders the material promising for non-invasive cardiac monitoring and wearable healthcare systems. However, long-term mechanical durability, signal stability, and performance under sweating and repetitive body movement need further assessment. Wang et al. fabricated a multifunctional ultrasensitive carbonize cellulose acetate (CCA)/TPU nanofiber membrane pressure sensor [82]. CCA provides conductive carbonized nanofibers, while TPU improves flexibility and reduces the brittleness of the carbonized network. The sensing mechanism is based on piezo resistive contact resistance changes between conductive nanofibers during pressure loading and unloading. This sensor can detect very small pressure changes, physiological signals, body movements and impact forces (Figure 7c). Owing to its high sensitivity, fast response, and excellent cycling durability, it can be used not only for healthcare monitoring but also for impact protection and safety warning systems. The combination of natural cellulose-derived carbon and TPU also supports and the development of lightweight and flexible smart textiles. However, challenges remain including the carbonization adds processing complexity, and the brittle carbonized precursor requires careful TPU reinforcement. Zhao et al. presented a high-precision facial expression recognition system based on the triboelectric hydrogel sensors and deep learning [83]. This work is relevant to smart healthcare monitoring, although the main sensing material is a hydrogel, as it demonstrates the potential of soft wearable sensors for facial movement detection, mental health monitoring, and human–computer interaction. The sensor generates electric signals from facial movement through the triboelectric effect without requiring an external power supply. These signals are processed by a 1D convolutional neutral network to classify facial expressions with high accuracy. This system can help monitor emotional status, support virtual medical communication and protect privacy compared with the camera-based facial recognition. The advantages include self-powered sensing, transparency, flexible, and high recognition accuracy. However, the dehydration of hydrogel and the difficulties in incorporating the sensor network are still major challenges for long-term use [83].

## 4. Conclusions and Challenges

TPU has shown great promise as a biomaterial for wound healing and smart healthcare applications owing to its excellent flexibility, elasticity, mechanical strength, biocompatibility, and tunable physicochemical properties. Its unique segmented structure enables the fabrication of multifunctional materials for a wide range of biomedical applications. Recent studies have demonstrated the broad potential of TPU in biomedical applications, including wound dressings, scaffolds for skin regeneration, tissue engineering constructs, and wearable healthcare devices. The incorporation of conductive nanomaterials, antibacterial agents, bioactive molecules, and self-healing functionalities has further enhanced the performance and versatility of TPU-based systems. These developments highlight the potential of TPU as a promising material for next-generation wound management, regenerative medicine, and real-time physiological monitoring applications.

However, several challenges must still be addressed before the widespread clinical translation of TPU-based biomedical systems can be achieved. Achieving the appropriate balance of mechanical robustness, flexibility, biodegradability, and biological functionality within a single material system remains a major challenge. The long-term biocompatibility, degradation behavior, and potential toxicity of TPU-based wound dressings also require further investigation. In addition, current TPU-based wound dressings often exhibit limited responsiveness to dynamic wound microenvironments, including infection, inflammation, pH changes, and excessive exudate production. Similarly, in smart healthcare applications, maintaining stable sensing performance, electrical conductivity, skin adhesion, and durability under continuous mechanical deformation remains challenging. Additional challenges, including large-scale manufacturing, sterilization compatibility, reproducibility, regulatory approval, and limited long-term clinical validation, must also be overcome. Therefore, further interdisciplinary research and clinical studies are needed to address these limitations and fully realize the potential of TPU-based technologies in advanced healthcare applications.

## 5. Future Perspectives

In the future, research on TPU is expected to focus on the design of multifunctional and smart materials that integrate wound healing, tissue regeneration, drug delivery, and physiological monitoring into a single platform. The development of biodegradable and bio-based TPU materials derived from renewable sources presents a promising strategy to improve sustainability while maintaining excellent mechanical and biological properties. Next-generation TPU dressings are expected to possess stimuli-responsive properties that enable dynamic responses to changes in the wound microenvironment, including pH, temperature, bacterial infection, and inflammatory signals, thereby facilitating more effective and personalized treatment. In skin regeneration and tissue engineering, the combination of TPU with bioactive molecules, growth factors, stem cells, ECM components, and functional nanoparticles is expected to enhance cellular responses and promote tissue repair. Advances in fabrication techniques, such as electrospinning, 3D printing, and 4D printing, will enable the production of patient-specific scaffolds and personalized biomedical devices with precise structural and functional features. Continuous, noninvasive health monitoring for smart healthcare applications is expected to be greatly advanced by TPU-based flexible electronics and wearable sensors. Future systems may integrate TPU-based sensing platforms with wireless communication technologies, AI, and the IoT to provide real-time health assessment and early disease diagnosis. Furthermore, the incorporation of self-powered functions may enhance device durability, reliability, and user comfort. Overall, ongoing advances in materials engineering, biomedical science, and digital health technologies are expected to further establish TPU as a key material for next-generation personalized healthcare and regenerative medicine applications.

## Figures and Tables

**Table 2:** Recent advances in TPU-based antibacterial and wound healing systems.

Authors	Materials	Application	Mechanisms	Methods	Advantages	Limitations	Ref.
Saghebal et al., 2023	PU-based nanofibrous mat	Wound healing with antibacterial and photodynamic therapy	Porphyrin activation, reactive oxygen species (ROS) generation, bacterial killing, and enhanced wound healing	In vitro antibacterial assays and an in vivo rat model	Excellent antibacterial activity, drug delivery capability, and biocompatibility	Reduced mechanical strength after additive incorporation and limited light penetration depth	[51]
Cheng et al., 2024	MXene/TPU hybrid	Smart wound dressing and drug delivery	Joule heating-induced temperature increase, controlled drug release, and wireless sensor-based monitoring	Electrospinning, spraying, and in vitro and in vivo wound models	Controlled drug release, real-time monitoring, and rapid wound healing	Complex system requiring device integration and associated high cost	[52]
Yildirim et al., 2024	Plasma-treated double-layer electrospun fiber mats	Wound dressing	Bilayer structure mimicking the epidermis and dermis; Hypericum perforatum oil provides antibacterial activity	Electrospinning, plasma treatment, and in vitro cell assays	Biomimetic structure and excellent antibacterial activity	Limited antimicrobial spectrum, lack of antifungal activity, and multistep fabrication	[53]
Li et al., 2024	Bioactive citrate-based PU	Tissue sealing and wound healing	Covalent bonding, hydrogen bonding, mechanical interlocking, and hydrophobic interactions	In vitro and in vivo wound healing studies	Strong wet adhesion, rapid tissue sealing, biocompatibility, and promotion of angiogenesis	Complex synthesis	[54]
Wang et al., 2024	Gallic acid-based PU	Smart materials, coatings, and wearable electronics	Dynamic phenol–carbamate bonds, self-healing, and thermoresponsive shape-memory behavior	Chemical synthesis and MOF incorporation	Self-healing, antimicrobial activity, shape-memory behavior, and recyclability	Challenges in MOF dispersion and the requirement for thermal activation	[55]
Zhao et al., 2024	Amphiphilic nanofibrillated cellulose/PU	Antibacterial and antifouling catheter materials	Electrostatic interactions, hydrogen bonding, contact killing, and anti-adhesion to prevent biofilm formation	One-step PU synthesis and blending with Am-CNF	Self-healing, strong antibacterial activity, antifouling properties, and high durability	Complex composition and possible long-term in vivo stability issues	[56]
Guo et al., 2024	Janus waterborne PU with quaternary ammonium salts	Wound healing adhesive patch	Asymmetric functional-group distribution, adhesive upper surface, anti-adhesive lower surface, and electrostatic antibacterial activity	Emulsion drying and spontaneous phase separation	Strong wet adhesion, antibacterial activity, and promotion of wound healing	Precise fabrication control required and limited long-term adhesion stability	[57]
Xu et al., 2024	Phase-separated AgNWs in porous PU	Wearable and implantable bioelectronics	Phase separation creates a porous structure and a strain-adaptive conductive network	In situ phase separation and drop casting	Ultra-low percolation threshold, high conductivity, and strain-insensitive performance	Use of metal fillers and fabrication complexity	[58]
Wang et al., 2025	Bio-based PU elastomer	Surgical sutures for wound healing	Strong hydrogen bonding, asymmetric hard segments, high mechanical strength, and self-healing	Degradation and biocompatibility studies	High tensile strength, toughness, self-healing, biodegradability, and sustainability	Complex processing, need to balance strength and elasticity, and limited wet-adhesion function	[59]
Ding et al., 2025	Dual-component PU bioadhesive	Soft tissue wound repair	Chemical bonding to tissue and rapid curing to improve adhesion	Adhesion testing in an in vivo rat model	High adhesive strength, flexibility, biocompatibility, and rapid wound closure	Optimization of curing time, synthesis complexity, and potential component toxicity	[60]
Zhang et al., 2025	Shark skin-inspired hydrophobic-modified PU	Infected and exudative wound healing and bacterial capture	Hydrophobic layer (antifouling barrier) and hydrogel layer (exudate absorption)	Antibacterial assays, ROS assays, and an in vivo wound healing model	Multifunctionality, efficient bacterial blocking, promotion of angiogenesis and collagen deposition, and immune modulation	Complex multistep fabrication, less precise drug release control, and possible variability in hydrogel performance	[61]
Jing et al., 2025	PU dressing with ultrafast laser-induced micro/nanostructures (PU-MS)	Anti-infective wound dressing for the prevention of *S. aureus* infection and SIRS	Physical structuring to create micro/nanocavities and enhance drug loading, with sustained antibiotic release	Ultrafast laser direct writing, spatiotemporal regulation, in vitro antibacterial assays, and an in vivo rat wound model	Maintains PU properties, strong antibacterial activity, and prevention of systemic infection	Requirement for laser instrumentation, dependence on antibiotics, and limited intrinsic antibacterial activity	[62]
Wang et al., 2026	Janus nanofiber dressing composed of PAN and TPU	Acute and chronic wounds	Spatiotemporal drug release, antibacterial activity, anti-inflammatory effects, AME, and sequential wound healing	Electrospinning, antibacterial assays, cell studies, and an in vivo wound model	Dual-function controlled drug release and improved angiogenesis and re-epithelialization	Complex fabrication, need for drug-loading optimization, and stability concerns regarding herbal components	[63]

**Table 3:** Recent advances in TPU-based materials for skin regeneration and tissue engineering applications.

Authors	Materials	Application	Mechanism	Methods	Advantages	Limitations	Ref.
Mrowka et al., 2023	HNT-filled TPU nanocomposites	Skin regeneration (post-skin cancer surgery)	HNT reinforcement improves mechanical properties and promotes selective cellular responses	Extrusion, cytotoxicity assays, and inflammatory assays	Improved mechanical properties, low toxicity toward healthy cells, and potential anticancer effects	Potential inflammatory response	[64]
Akkurt Yildirim et al., 2023	CLN-doped TPU nanocomposites	Skeletal muscle tissue engineering scaffold	CLN enhances hydrogen bonding, cell adhesion, and cell proliferation while modifying the mechanical and thermal properties of the TPU scaffold	Electrospinning, tensile testing, swelling tests, biodegradation analysis, and MTT assays	Biocompatibility, enhanced cell adhesion and proliferation, tunable mechanical properties, good thermal stability, and suitable elasticity for muscle tissue	Particle agglomeration at high CLN loading, reduced hydrogen bonding at high CLN concentrations, lack of biodegradation, and reduced swelling capacity	[65]
Giubertoni et al., 2023	TPU with hydrogen-bonded urethane groups	Understanding the molecular origin of the mechanical properties of polymers	Strain-induced rearrangement of hydrogen bonds, increased hydrogen bonding at moderate strain, dissociation of weak hydrogen bonds at high strain, and narrowing of the hydrogen-bond distribution after deformation	Rheo-2D IR, conventional FTIR spectroscopy, and stress–strain analysis	Provides molecular-level insights, distinguishes homogeneous broadening, reveals structural changes not detected by conventional IR, and explains the Mullins effect	Complex and expensive instrumentation, requirement for spectroscopic expertise, limitation to thin-film samples, and time-consuming measurements	[66]
Yuvan et al., 2024	PU–PEGDA hydrogel with bioactive peptides	Tissue engineering scaffolds	UV photocrosslinking forms a three-dimensional network, while peptide incorporation enhances cell adhesion	Mechanical testing and cell viability assays	Tunable mechanical properties, enhanced cell adhesion, and potential for biofunctionalization	Limited PU solubility and the need for further optimization before clinical translation	[67]
Huang et al., 2024	PU elastomer with tendon ECM	Tendon regeneration	ECM provides biological cues, whereas PU provides mechanical support to promote tendon regeneration	Core–shell scaffold fabrication, mechanical testing, in vitro stem cell studies, and in vivo animal models	Biomimetic design, strong mechanical properties, excellent tissue integration, and support for large tendon defect repair	Complex fabrication, ECM variability, and cost and scalability issues	[68]
Hu et al., 2025	Polysiloxane-based PU	Wound dressing	Antimicrobial activity mediated by cationic groups, antifouling properties provided by zwitterionic components, and a bilayer structure that mimics skin	Antibacterial assays and cytocompatibility tests	Strong antimicrobial activity, antifouling properties, good moisture balance, and flexibility	Complex synthesis and potential long-term stability issues	[69]
Karahaliloglu et al., 2024	TPU–oleic acid membranes	Guided bone regeneration	Increased hydrophilicity and nanoscale surface roughness enhance cell adhesion and antibacterial activity	Solvent casting and antibacterial assays	Improved wettability, enhanced cell attachment, and antibacterial properties	Surface modification required and long-term durability not fully evaluated	[70]
Kordbacheh et al., 2025	Polycaprolactone (PCL)/TPU/barium titanate/cellulose nanocrystal composite	Bone tissue engineering (piezoelectric scaffold)	Mechanical stress generates electrical signals through the piezoelectric effect	Gas foaming, salt leaching, cell studies, and mechanical and electrical characterization	Self-stimulating properties, high porosity, good cell adhesion, and electrical signaling that mimics natural bone	Brittleness of barium titanate, polymer immiscibility, and low electrical output	[71]

**Table 4:** Summary of TPU-based real-time physiological monitoring system applications.

Authors	Materials	Application	Mechanism	Methods	Advantages	Limitations	Ref.
Ding et al., 2023	MXene/TPU nanofiber membrane	Humidity sensor (respiration and sleep monitoring)	Grotthuss proton transport mechanism	Electrospinning and sputtering	High sensitivity, rapid response, and breathability	MXene oxidation	[74]
Haridas et al., 2023	Recyclable TPU/graphene composite	Strain and pressure sensor	Percolation-based conductive network	Melt mixing	Recyclable, stable, and flexible	Moderate sensitivity	[75]
Ai et al., 2024	TPU/tetraphenylethylene (TPE)-plied yarn	Strain sensor for health monitoring	Crack propagation and conductive network variation (dual optical/electrical sensing)	Wet spinning and in situ polymerization	Very high sensitivity and dual-mode sensing	Complex fabrication	[76]
Jung et al., 2024	Thermally robust nanofibrous radiative cooler and strain-insensitive conductor	Outdoor wearable electronics and physiological monitoring	Strain-insensitive conductive mechanism and radiative cooling	Electrospinning and laser patterning	Excellent thermal stability, strain-insensitive performance, and operation under sunlight	Fabrication complexity and high material cost (liquid metal conductors)	[77]
Liu et al., 2024	MXene nanosheets and PU elastomer	Electronic skin (e-skin), healthcare monitoring, and human–machine interaction	Piezoresistive sensing and microstructure amplification	Spin coating, templating, and MXene coating	Ultra-high sensitivity (1573 kPa^−1^), self-healing, and antibacterial properties	Complex synthesis and thermal activation required for self-healing	[78]
Sun et al., 2024	AgNWs, single-layer graphene (SLG), and PU sponge	Wearable pressure sensing and human motion monitoring	Piezoresistive mechanism (changes in electrical resistance under applied pressure)	Dip coating and polyol synthesis	Low cost, flexibility, high reproducibility, and good durability (3000 cycles)	Moderate sensitivity and dependence on pore structure optimization	[79]
Cao et al., 2025	MXene/GO-based modified TPU	Basketball shooting posture monitoring (sports and EMG signals)	Hydrogen-bond-dominated conductive network and electron transport pathways	Solution mixing, integrated molding, and deep learning	High stability, wide strain-sensing range (0–240%), and AI integration	MXene stability issues and system complexity	[80]
Krishna Rajeev et al., 2025	Polyaniline (PANI)/MWCNT/ZnO-reinforced PU foam	ECG signal monitoring	Electrical signal conduction through a conductive polymer network	In situ polymerization and composite reinforcement	ECG signals comparable to those of Ag/AgCl electrodes, flexibility, and low cost	Mechanical durability and long-term stability concerns	[81]
Wang et al., 2025	Carbonized cellulose acetate (CCA)/TPU nanofiber membrane	Pressure sensing and physiological monitoring	Piezoresistive sensing (contact resistance changes within the conductive network)	Electrospinning, carbonization, and filtration	Very high sensitivity, rapid response, and durability	Carbonization complexity and brittleness of the carbonized network	[82]
Zhao et al., 2025	Triboelectric hydrogel/PDMS/LiCl	Facial expression recognition (FER) and mental health monitoring	Triboelectric effect and deep-learning-based signal processing	Hydrogel synthesis and sensor fabrication (1D-CNN)	Self-powered operation, high accuracy, flexibility, and transparency	Complex system and hydrogel dehydration	[83]

## Data Availability

No new data were created or analyzed in this study.
